# Case Report: A pediatric case of chronic active Epstein–Barr virus infection complicated by pulmonary arterial hypertension

**DOI:** 10.3389/fcvm.2026.1724841

**Published:** 2026-02-20

**Authors:** Meng Zhang, Kai Wang, Xinyi Xu, Wei Ji, Tingliang Liu, Wei Gao, Ying Guo

**Affiliations:** Department of Cardiology, Shanghai Children’s Medical Center, School of Medicine, Shanghai Jiao Tong University, Shanghai, China

**Keywords:** cardiovascular damage, child, chronic active EBV infection, orphan disease, pulmonary arterial hypertension

## Abstract

**Objective:**

To enhance clinicians' awareness of pulmonary arterial hypertension (PAH) complicating chronic active Epstein–Barr virus infection (CAEBV) in pediatric patients.

**Method:**

Clinical data of a pediatric patient diagnosed with CAEBV complicated by PAH, admitted to Shanghai Children's Medical Centre affiliated with Shanghai Jiao Tong University School of Medicine, were analyzed.

**Results:**

The patient, a 11-year-old girl, was admitted with “dyspnea on exertion accompanied by left lower abdominal pain for 1 month.” The primary clinical manifestations constituted progressive decline in exercise tolerance and abdominal pain. Physical examination revealed hepatosplenomegaly and scattered eczematous rashes on the left lower limb. Cardiac ultrasound demonstrated moderate tricuspid regurgitation with a velocity of 3.56 m/s and estimated pulmonary artery pressure of 50 mm Hg. The outpatient department considered PAH and referred her to cardiology. Right heart catheterization upon admission revealed pulmonary artery pressure of 81/56/67 mm Hg, synchronous aortic pressure of 121/76/92 mm Hg, and pulmonary vascular resistance index (PVRI) of 24 Wood, confirming PAH. During comprehensive screening for PAH etiology, markedly abnormal EBV antibody levels were identified, and levels of EBV-DNA in the peripheral were significantly increased, raising the suspicion of chronic active Epstein–Barr virus infection (CAEBV). Subsequent skin biopsy of the left-lower-limb lesion demonstrated EBER positivity, confirming diagnosis of CAEBV with PAH. Allogeneic hematopoietic stem-cell transplantation was recommended after diagnosis but declined by the family. Eight months later, EBV had disseminated to multiple systemic organs. Cerebrospinal fluid tested positive for EBV nucleic acid; bone marrow biopsy showed partial EBER positivity; hepatosplenomegaly was present; and concomitant conditions included abdominal wall varicosities, multiple serosal effusions, hypoxemia, and PAH. Upon readmission, the patient's baseline condition deteriorated to the point where allogeneic hematopoietic stem-cell transplantation was no longer feasible. Conservative chemotherapy was initiated; however, the patient succumbed to septic shock complicated by gastrointestinal hemorrhage one month into treatment.

**Conclusion:**

The etiology of PAH is complex and diverse, with CAEBV representing a rare causative factor. Comprehensive EBV-related pathogen testing should be performed in children with PAH, particularly those presenting with hepatosplenomegaly. Routine imaging investigations, including cardiac echocardiography, are essential in the assessment and management of CAEBV patients to promptly identify potential cardiovascular complications.

## Introduction

Chronic active Epstein–Barr virus infection (CAEBV) is a rare disease mainly caused by clonal proliferation of T cells or NK cells infected with EBV, which can be oligoclonal, monoclonal, and polyclonal proliferation, accompanied by persistent EBV infection leading to disease onset. CAEBV is mainly seen in pediatric patients (1). It is characterized by various symptoms similar to infectious mononucleosis, such as fever, liver damage, and hepatosplenomegaly, but it often leads to serious complications, such as hemophagocytic syndrome, disseminated intravascular coagulation, interstitial pneumonia, and liver failure. Cardiovascular-related complications caused by CAEBV are generally rare. Among them, coronary artery aneurysms and myocarditis are more common, while pulmonary arterial hypertension (PAH) caused by CAEBV is even rarer, with less than 10 cases reported globally. Here, we report a rare case of a patient admitted to Shanghai Children's Medical Center with the onset of PAH, ultimately diagnosed as CAEBV, aiming to improve physicians' understanding of the related cardiovascular damage caused by CAEBV. This study was approved by the Medical Ethics Committee of Shanghai Children's Medical Center (Approval number: SCMCIRB-K202226-1), exempting guardians from informed consent.

## Case report

**Figure 1:**
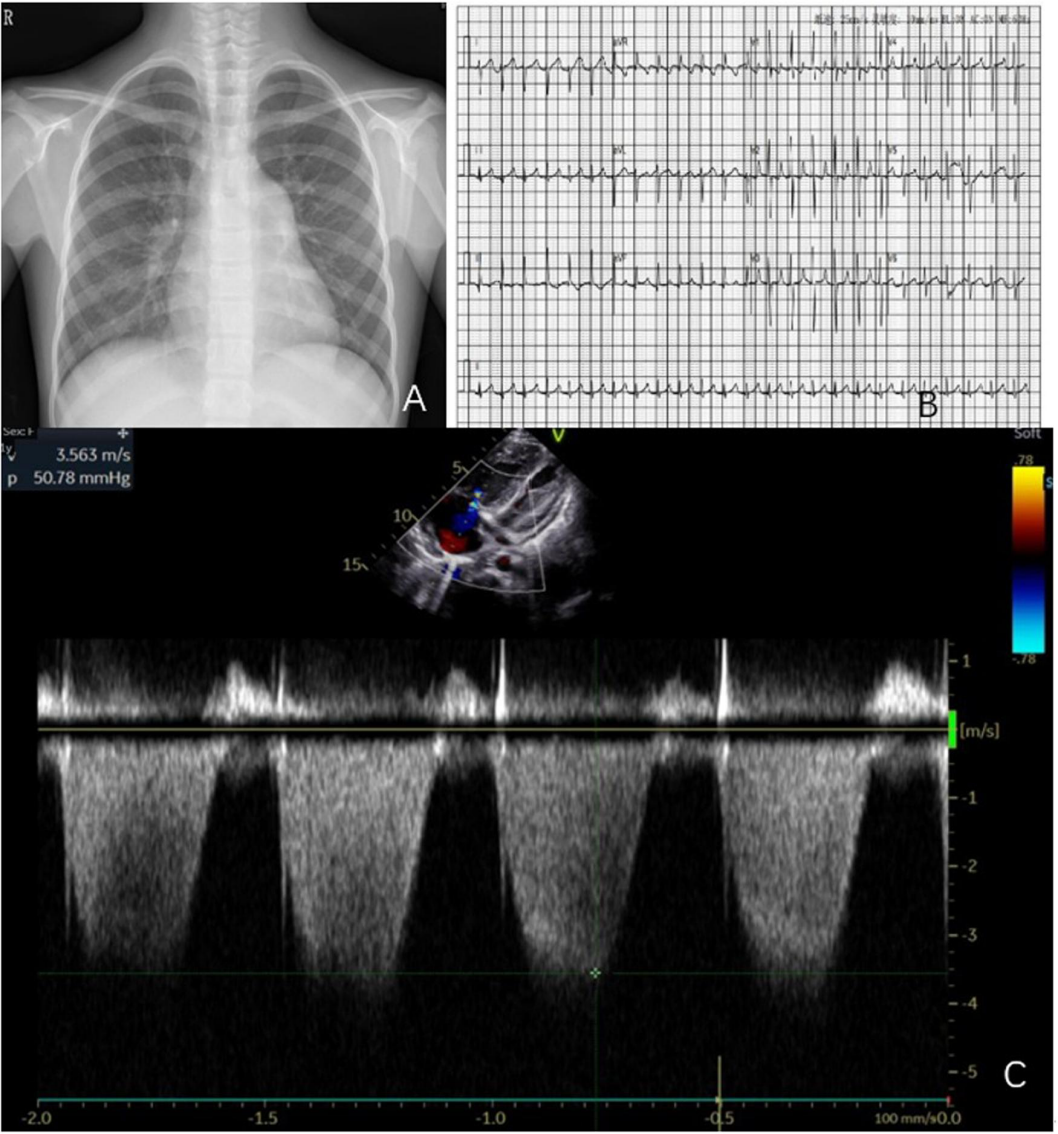
**(A–C)** Chest radiograph, electrocardiogram, and ultrasound images of the patient, with cardiac ultrasound indicating right-atrial and right-ventricular enlargement, and tricuspid regurgitation velocity of 3.56 m/s, with estimated pulmonary artery pressure of 50 mm Hg.

A Chinese female child aged 11 years and 1 month was diagnosed with “decreased activity tolerance accompanied by abdominal pain for 1 month” at a nearby hospital. A complete echocardiogram showed moderate tricuspid regurgitation, reverse flow velocity of 3.56 m/s, and estimated pulmonary artery pressure of 50 mm Hg. After 1-month treatment with bosentan, the symptoms worsened and the patient was referred to the Cardiovascular Department of Shanghai Children's Medical Center. The child had a negative medical history, no history of trauma or surgery, no family history of genetic disorders, and no history of drug or toxin use. At admission, body temperature was 37.3 °C, pulse rate was 138/minute, respiration rate was 24/minute, blood pressure was 92/61 mm Hg (1 mm Hg = 0.133 kPa), and body weight was 31.2 kg. The heart sounds were strong, with the second heart sounds being particularly loud in the pulmonary artery auscultation area. The liver and spleen were enlarged without tenderness. There was no edema in both lower limbs, and an eczema-like rash was visible in the left lower limb. A chest radiograph and an electrocardiogram are shown in Figures 1A,B. Echocardiography evaluation showed tricuspid regurgitation velocity of 3.56 m/s, with estimated pulmonary artery pressure of 50.7 mm Hg, as shown in Figure 1C.

**Figure 2:**
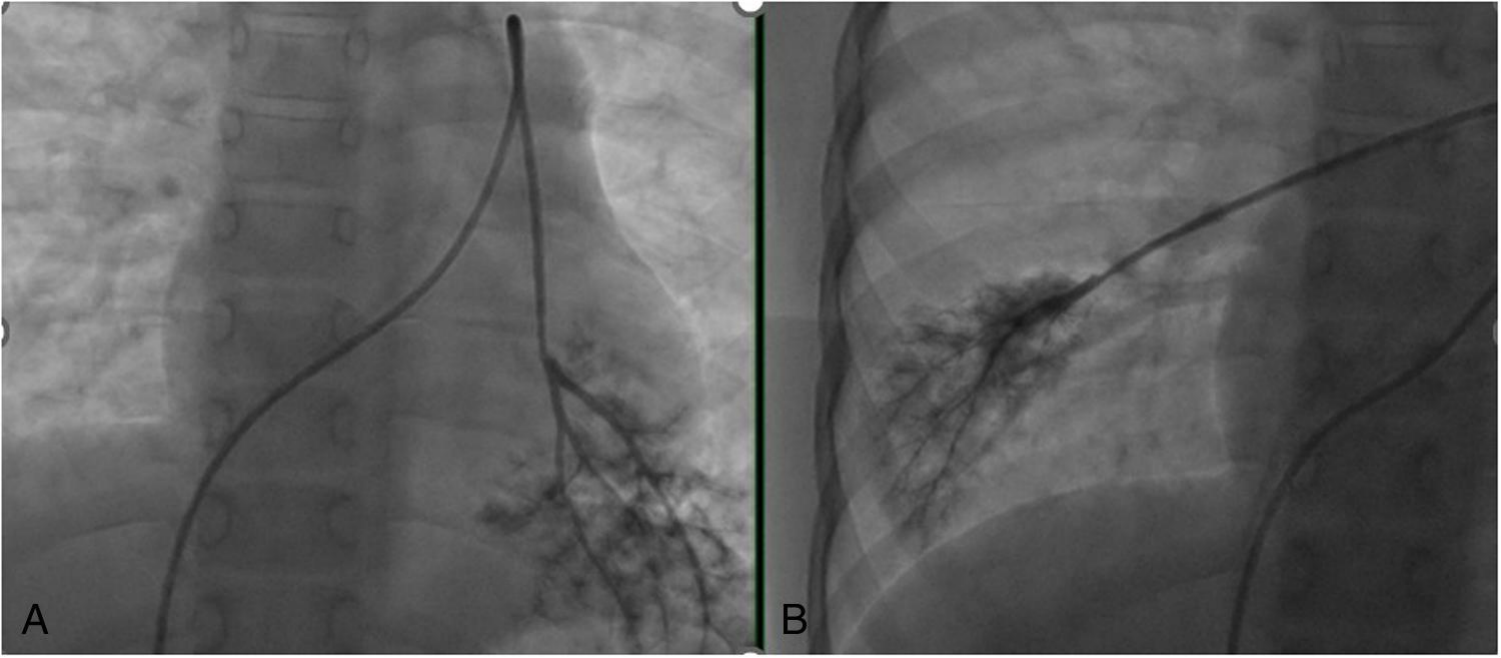
**(A,B)** Pulmonary arteriole compression angiography images.

After admission, a complete right heart catheterization was performed, showing right-atrial pressure of 14 mm Hg, pulmonary artery pressure of 81/56/67 mm Hg, synchronous aortic pressure of 121/76/92 mm Hg, calculated pulmonary small-artery resistance (PVRI) of 10.8 Wood, and pulmonary small artery morphology illustrated in Figures 2A,B. The routine laboratory tests after admission are shown in Table 1. In addition, the results of rheumatoid immune antibodies such as anti-nuclear antibody spectrum, In addition, the results of the relevant rheumatic and immune antibodies for this child were negative, as were the results for common pathogens (such as hepatitis viruses, syphilis, CMV, and HIV). Blood urine tandem mass spectrometry was also negative.

**Table 1:** Routine laboratory test results after admission.

Parameter	Numerical value	Reference range
Leukocytes	4.81 × 10^9^/L	4–10 × 10^9^/L
Platelets	98 × 10^9^/L	100–300 × 10^9^/L
Hemoglobin	120.0 g/L	120–160 g/L
CRP	10 mg/L	0–8 mg/L
ESR	29 mm/hour	0–20 mm/hour
NT-pro BNP	96 pg/mL	0–250 pg/mL
AST	23 U/L	5–35 U/L
ALT	36 U/L	0–40 U/L
Total bilirubin	7.7 µmol/L	5–19 µmol/L
Albumin	32.0 g/L	35–55 g/L
Creatinine	<29 µmol/L	<29 µmol/L
DIC	-	-

**Figure 3:**
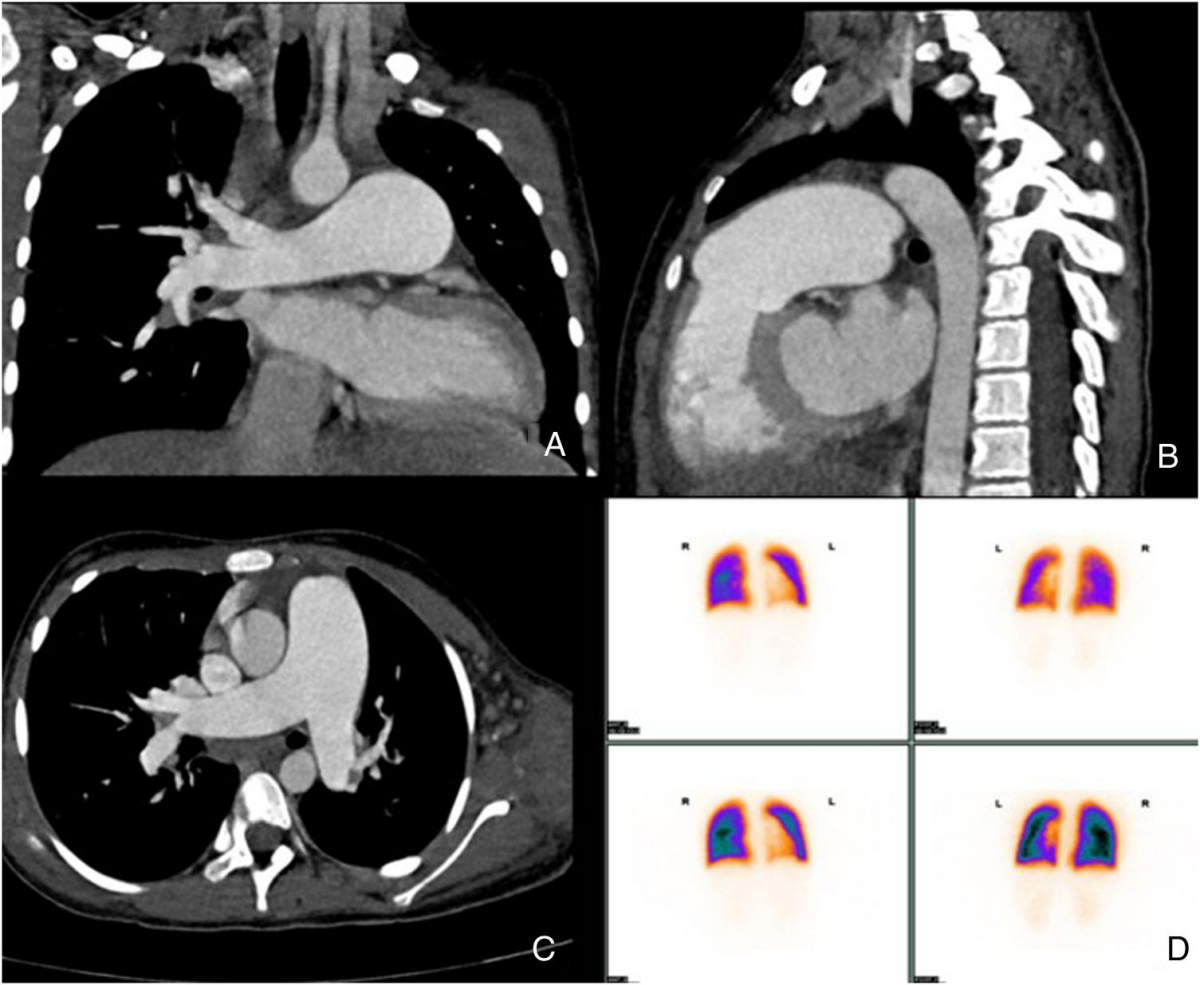
**(A–C)** Pulmonary artery enhanced CT shows total pulmonary artery dilation; **(D)** shows pulmonary perfusion imaging, demonstrating normal ratio of the left and right lung blood flow.

The results of cardiac enhanced CT and nuclear pulmonary ventilation/perfusion imaging showed no significant abnormalities, and no pulmonary hypertension caused by chronic thromboembolic pulmonary hypertension or pulmonary artery obstructive lesions was found. The signs of pulmonary developmental diseases, restrictive pulmonary diseases, pulmonary vein occlusion, and other related diseases were also excluded (Figures 3A–C). Cardiac MRI revealed pulmonary artery dilation, right-atrium and right-ventricle enlargement, and normal right-ventricular systolic function. The whole-exome gene test was negative.

**Figure 4:**
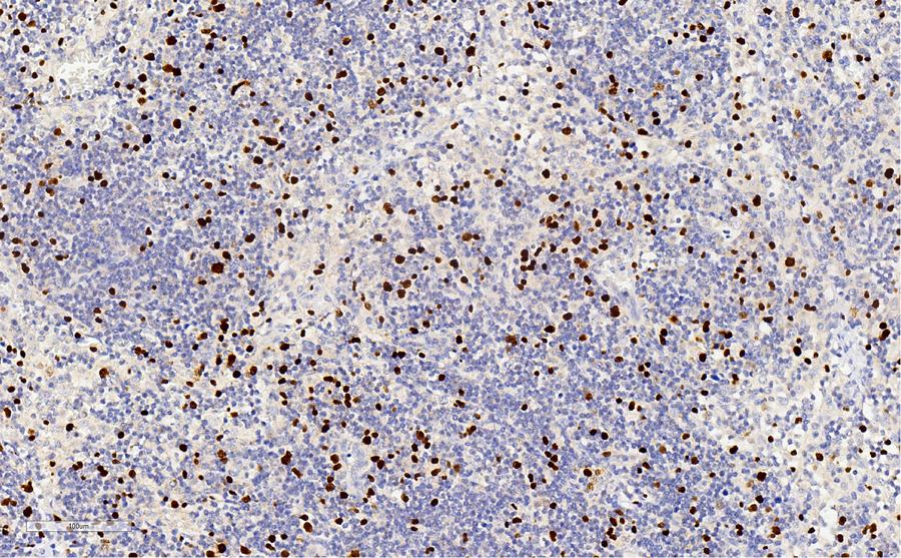
Pathological biopsy: EREB positivity.

However, anti-EBV antibody titers were significantly abnormal, with anti-EBV capsid antibody IgG > 750.00 U/mL, early anti-EBV antibody IgG > 150.00 U/mL, and anti-EBV nuclear antibody IgG 315.00 U/mL. Combined with the significant enlargement of the spleen and eczema-like lesions on the left lower limb, further peripheral-blood EBV detection, bone marrow puncture, and skin pathological biopsy were performed. The results were as follows: blood EBV-DNA quantification: 3.33 × 10^4^ copies/mL; EBV-TDNA: 2.37 × 10^6^ copies/mL; EBV-BDNA: 5.63 × 10^5^ copies/mL; and EBV-NK DNA: 6.41 × 10^4^ copies/mL. All of these results are significantly abnormal. Bone marrow examination showed no signs of hemophagosis. Pathological biopsy of the damaged skin on the left lower limb suggested mild proliferation and degeneration of the dermal fibrous tissue, scattered lymphocyte infiltration, and more important result was EBER+, as shown in Figure 4. After considering the pathological results, the following diagnoses were made: CAEBV, PAH (severe, moderate risk), and grade II heart failure. Allogeneic hematopoietic stem-cell transplantation was recommended, but the family refused. After discharge, treatment with acyclovir, bosentan, and sildenafil was administered.

**Figure 5:**
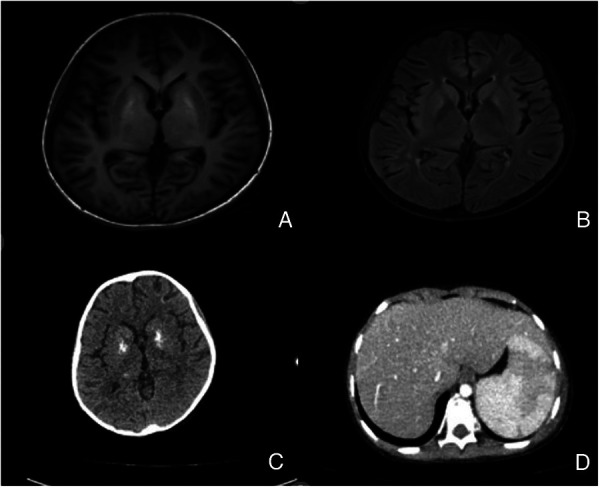
**(A,B)** T1 and T2 FLAIR imaging suggest the absence of the basal ganglia on both sides, and there are multiple abnormal signals near the ventricles on both sides.); **(C)** head CT scan shows multiple calcifications in the basal ganglia and ventricles on both sides, suggesting viral infiltration. **(D)** Abdominal enhanced CT shows extensive infiltration of the spleen.).

Eight months later, the child was admitted to the hospital due to “recurrent headaches and shortness of breath for several days.” Admission physical examination revealed body temperature of 37.5 °C, pulse rate of 150/minute, respiration rate of 30/minute, blood pressure of 94/75 mm Hg, SpO_2_ of 91% without oxygen inhalation, decreased respiratory sounds in both lungs, and increased second heart sounds in the pulmonary artery auscultation area. Abdominal distension, hepatosplenomegaly, superficial varicose veins on the abdominal wall, eczema-like rash on the left lower limb, and palpable mass on the left shoulder were also noted. The patient's basic condition was poor upon admission, after which a systematic evaluation was conducted. The results were as follows:

Circulatory system: Cardiac ultrasound revealed moderate tricuspid regurgitation with a reflux velocity of 3.60 m/s and estimated pulmonary artery pressure of 52 mm Hg. NT pro-BNP level was 498 pg/mL. The disease worsened in the later stage, with NT pro-BNP levels increasing to 2,070 pg/mL. The quantitative changes of peripheral-blood EBV, pulmonary artery pressure, and NT pro-BNP levels are shown in Table 2.
Nervous system: Cerebrospinal fluid tested positive for EBV nucleic acid. Further cerebrospinal fluid examination revealed the following findings: chlorine, 124 mmol/L; glucose, 1.8 mmol/L; protein, 562 mg/L; white blood cells, 1 × 10^6^/L. Head MRI showed patchy T1WI low signal, and T2WI and FLAIR high signal shadows in the basal ganglia on both sides of the ventricles (as shown in Figures 5A–C), suggesting EBV encephalitis.
Respiratory system: Chest CT showed moderate pleural effusion on both sides, accompanied by pulmonary infection and respiratory distress.
Digestive system: Abdominal ultrasound and CT indicated significant enlargement of the liver and spleen, with widespread viral infiltration of the spleen (Figure 5D), widened portal and splenic veins, and abdominal fluid accumulation.
Hematological system: Bone marrow biopsy indicated EBER-positivity, and the level of EBV-DNA in the blood gradually increased.

**Table 2:** Changes in peripheral-blood EBV quantification, pulmonary artery pressure, and NTpro BNP.

date	Quantification of EBV-DNA in peripheral blood, copies/mL	Tricuspid regurgitation velocity, m/s	Pulmonary artery pressure, mmHg	NT pro-BNP, pg/mL
2022.9.9	3.33 × 10^4^	3.56	50.8	96
2022.9.16	2.56 × 10^4^	4.07	66	499
2022.11.4	4.78 × 10^4^	3.67	54	588
2023.3.5	1.25 × 10^4^	3.8	58	873
2023.6.26	4.81 × 10^5^	3.6	52	979
2023.7.14	9.85 × 10^5^	3.31	44	2,070

Given that EBV spread to multiple systems throughout the body, with the patient's poor baseline condition and inability to tolerate transplantation, conservative chemotherapy was administered. The patient died 1 month later due to disease progression and gastrointestinal bleeding.

## Discussion

Since Horwitz (2) reported the world's first case of CAEBV in 1975, its pathogenesis has remained unknown. Mutations in the perforin gene have been detected in some of the CAEBV cases. The overall prognosis is extremely poor, and CAEBV can lead to life-threatening complications, such as hemophagocytic syndrome, malignant lymphoma, disseminated intravascular coagulation, and liver failure (3). However, cardiovascular complications are relatively rare. According to the 2017 WHO classification, nearly 10% of CAEBV patients may have concurrent cardiovascular problems (4). Based on the diagnostic criteria for CAEBV (5), the diagnosis of CAEBV in this case was clear. Previously, Yonese et al. (1) found that about 7% of CAEBV patients developed vasculitis, while 9% of patients had late-stage manifestations of heart failure. Coronary artery aneurysm and myocarditis are the main cardiovascular complications of CAEBV in pediatric patients, and CAEBV leading to PH is extremely rare. In 2011, Hashimoto et al. (6) reported the first adult case of CAEBV combined with PAH. In 2015, Fukuda et al. (7) reported the first case of CAEBV combined with PAH in a child. Hitherto, a total of 13 cases have been reported worldwide, including 11 adults and 2 children, among whom 9 died and 4 survived.

At present, the specific causes of cardiovascular damage by CAEBV are not clear, and the potential mechanisms include direct and indirect injuries. Initially, Nakagawa et al. (8) performed postmortem examination of a pediatric patient with a massive coronary artery aneurysm caused by EBV infection, revealing lymphocyte infiltration and EBER-positivity at the site of the coronary artery lesion. Ba and others (9) found EBER-positive cells in patients with CAEBV combined with PH. However, in a 45-year-old adult patient, Hashimoto and others (6) did not find EBER-positive cells in the lung tissue. Misaki (10) reported on four adult patients with CAEBV and PH, stating that EBER-positive cells were found in the lung tissue of two of them. However, fibrosis of pulmonary small blood vessels was observed in various pathological sections. Accordingly, it was speculated that, in CAEBV, continuous damage to various types of lymphocytes infected with EBV induces the production of fibrin-like arteritis, leading to constrictive lesions such as thickening of the intima, media, and adventitia of pulmonary arteries, and subsequently forming complex lesions such as PAH intrinsic plexiform and expansive lesions.

The patient in this case resembles previously reported cases (6, 7, 10). The clinical presentation was insidious, without typical infectious mononucleosis-like symptoms; instead, nonspecific symptoms such as shortness of breath after activity dominated the presentation. According to the guidelines for pulmonary hypertension (11), PAH caused by CAEBV should be classified as the fifth of the five major etiological categories of pulmonary hypertension, namely PAH caused by unknown/multifactorial factors. Due to the low incidence rate, it is often easy to miss this diagnosis. This further indicates that the etiology of pulmonary hypertension is complex, and newly diagnosed patients should undergo comprehensive EBV-related testing. At the same time, in the evaluation and diagnosis of CAEBV patients, routine imaging examinations such as cardiac ultrasound should be performed to timely detect the presence of complications such as cardiovascular damage.

In our case, there was no correlation between EBV peripheral-blood DNA quantification and pulmonary arterial hypertension. The higher the EBV-DNA quantification in peripheral blood, the less severe the pulmonary arterial hypertension. For PH caused by CAEBV, the treatment with pulmonary vasodilators may play an important role. In our case, pulmonary artery pressure did not continue to increase in the later stage of the disease. Misaki and others (10), Akagi et al. (12), and Onishi et al. (13) reached similar conclusions.

However, the ultimate treatment remains targeted at CAEBV itself. In recent years, multiple therapeutic approaches have been developed for CAEBV, with the current standard being a “three-step therapy”: immunochemotherapy, followed by multi-agent combination chemotherapy, culminating in allogeneic hematopoietic stem-cell transplantation. With conventional chemotherapy regimens alone (such as modified Kan-Escape protocols), achieving complete remission in pediatric patients remains challenging. Combination therapies incorporating immunosuppressants such as sirolimus, lenalidomide, and ruxolitinib represent novel approaches for treating CAEBV. Previous studies (14, 15) have attempted various chemotherapy regimens, which improved peripheral blood EBV viral load and pH post-treatment, yet disease recurrence occurred upon discontinuation. Allogeneic hematopoietic stem-cell transplantation is currently the only recognized curative approach for CAEBV. Recently, Liu et al. (16) have reported a case of CAEBV with predominant gastrointestinal manifestations successfully treated by CAR-T therapy, demonstrating promising prospects for CAR-T therapy in CAEBV management. In the present case, during the second hospitalization, the primary treatment challenge was no longer PAH but multiorgan dysfunction caused by EBV dissemination, highlighting the disease's rapid progression and inherent danger.

Overall, given the complex and diverse etiology of PAH, EBV screening should be incorporated into diagnostic and therapeutic considerations. While PAH arising from CAEBV is rare, comprehensive cardiac investigations should be pursued early in such pediatric patients to prevent misdiagnosis or missed diagnosis.

## Data Availability

The raw data supporting the conclusions of this article will be made available by the authors, without undue reservation.
